# A scoping review of mathematical modeling techniques for gingival keratinization: A framework for periodontal research

**DOI:** 10.4317/jced.62834

**Published:** 2025-10-01

**Authors:** Pradeep Kumar Yadalam, Raghavendra Vamsi Anegundi, Carlos M Ardila

**Affiliations:** 1 Department of Periodontics, Saveetha Dental College and Hospital, Saveetha Institute of Medical and Technical Sciences, Saveetha University, Chennai,Tamil Nadu, India; 2 Basic Sciences Department. Biomedical Stomatology Research Group, Faculty of Dentistry Universidad de Antioquia, UdeA, Medellín, Colombia

## Abstract

**Background:**

Gingival keratinization is a critical physiological process that protects against mechanical stress and microbial invasion. Disruptions in this process contribute to periodontal diseases, affecting over 50% of adults worldwide. Despite its clinical significance, the molecular mechanisms of gingival keratinization remain poorly understood. This scoping review evaluates three predominant mathematical modeling paradigms—Gene Regulatory Networks (GRNs0, Ordinary Differential Equations (ODEs), and Agent-Based Models (ABMs)—to establish a framework for periodontal research.

**Material and Methods:**

A comprehensive literature search was conducted in PubMed, Web of Science, and IEEE Xplore, identifying 42 studies for analysis. Models were assessed across six dimensions: biological scale, spatial-temporal resolution, stochasticity, computational complexity, and perturbation response. Quantitative scoring was applied to compare capabilities in gene expression, temporal dynamics, and spatial modeling. Statistical analysis included one-way ANOVA and Tukey’s HSD test.

**Results:**

ABMs demonstrated superior versatility (total score: 75.0%) in simulating spatial organization and mechanical stress responses, while GRNs excelled in gene expression modeling (score: 9/10) and ODEs in temporal dynamics (score: 7/10). Perturbation coverage was highest for ABMs (87.5%), particularly for inflammation and mechanical stress. GRNs and ODEs scored 62.1% and 65.2%, respectively, with strengths in genetic and population-level dynamics.

**Conclusions:**

ABMs are optimal for spatial and stochastic modeling, whereas GRNs and ODEs are better suited for molecular and temporal analyses. Integrating these approaches could provide a comprehensive understanding of gingival keratinization. This review offers guidelines for model selection based on research objectives and computational resources.

** Key words:**Gingival keratinization, Periodontal diseases, Gene regulatory networks, Differential equations, Computational biology, Systems biology.

## Introduction

Gingival keratinization is a critical physiological process that safeguards oral health by forming a protective barrier against mechanical stress, microbial invasion [1,2], and chemical irritants [3]. This specialized epithelial differentiation produces keratinized gingiva, a stratified squamous epithelium with regionally variable keratinization patterns surrounding the alveolar bone and teeth. Disruptions in this process contribute to prevalent periodontal conditions, including gingival recession, peri-implantitis, and inflammatory periodontal diseases, which affect over 50% of adults worldwide. Despite its clinical significance, the molecular and biomechanical mechanisms governing gingival keratinization remain incompletely elucidated, particularly the crosstalk between epithelial-mesenchymal interactions, inflammatory mediators, and mechanical forces during homeostasis and disease.

The width and quality of keratinized gingiva are key determinants of periodontal health, with insufficient keratinization linked to heightened susceptibility to inflammation and tissue breakdown [4,5]. Keratinized tissue acts as a first line of defense, and its loss exacerbates pathogen penetration and immune dysregulation. Current research employs diverse methodologies—histology, 3D bioprinting, cell cultures, and biochemical assays—yet mathematical modeling has emerged as a powerful tool to unravel the multi-scale dynamics of keratinization, from gene regulation to tissue-level architecture.

Mathematical models enable predictive simulations of biological parameters, offering insights into therapeutic interventions and pathological conditions [6,7]. These models bridge gaps between experimental observations and clinical outcomes by quantifying the interplay of molecular drivers (e.g., transcription factors, signaling pathways) and macroscopic phenotypes. Prior studies have leveraged Gene Regulatory Networks (GRNs) to decode keratinocyte differentiation dynamics [8], Ordinary Differential Equations (ODEs) to simulate epithelial turnover [7], and Agent-Based Models (ABMs) to explore spatial tissue organization [9-11]. For instance, GRN frameworks have revealed how perturbations in keratin production or degradation alter network stability [7], while lipid-mediated regulatory models highlight metabolic influences on differentiation timing [6,9,10]. However, these approaches often lack integration of stress responses or spatial heterogeneity, limiting their translational relevance.

Notably, the KEAP1/NRF2 pathway exemplifies the role of redox signaling in keratinization, where NRF2 activation promotes cytoprotective gene expression but requires quantitative thresholds for clinical targeting [5]. Similarly, ABMs excel in simulating 3D epidermal self-organization [11] yet often omit intracellular signaling dynamics, hindering mechanistic interpretations. While GRNs focus on genetic circuits, ODEs predict population-level behaviors, and ABMs capture emergent tissue architectures [12-15], no systematic comparison has evaluated their suitability for gingival keratinization-specific challenges, such as regional variations (attached vs. marginal gingiva) or microbiome interactions.

This scoping review addresses this gap by rigorously comparing GRN, ODE, and ABM paradigms across six dimensions: biological scale, spatial-temporal resolution, stochasticity, computational complexity, perturbation response, and clinical applicability. Our goal is to provide a decision-making framework for periodontal researchers, balancing biological fidelity with computational feasibility.

## Material and Methods

**Figure 1:**
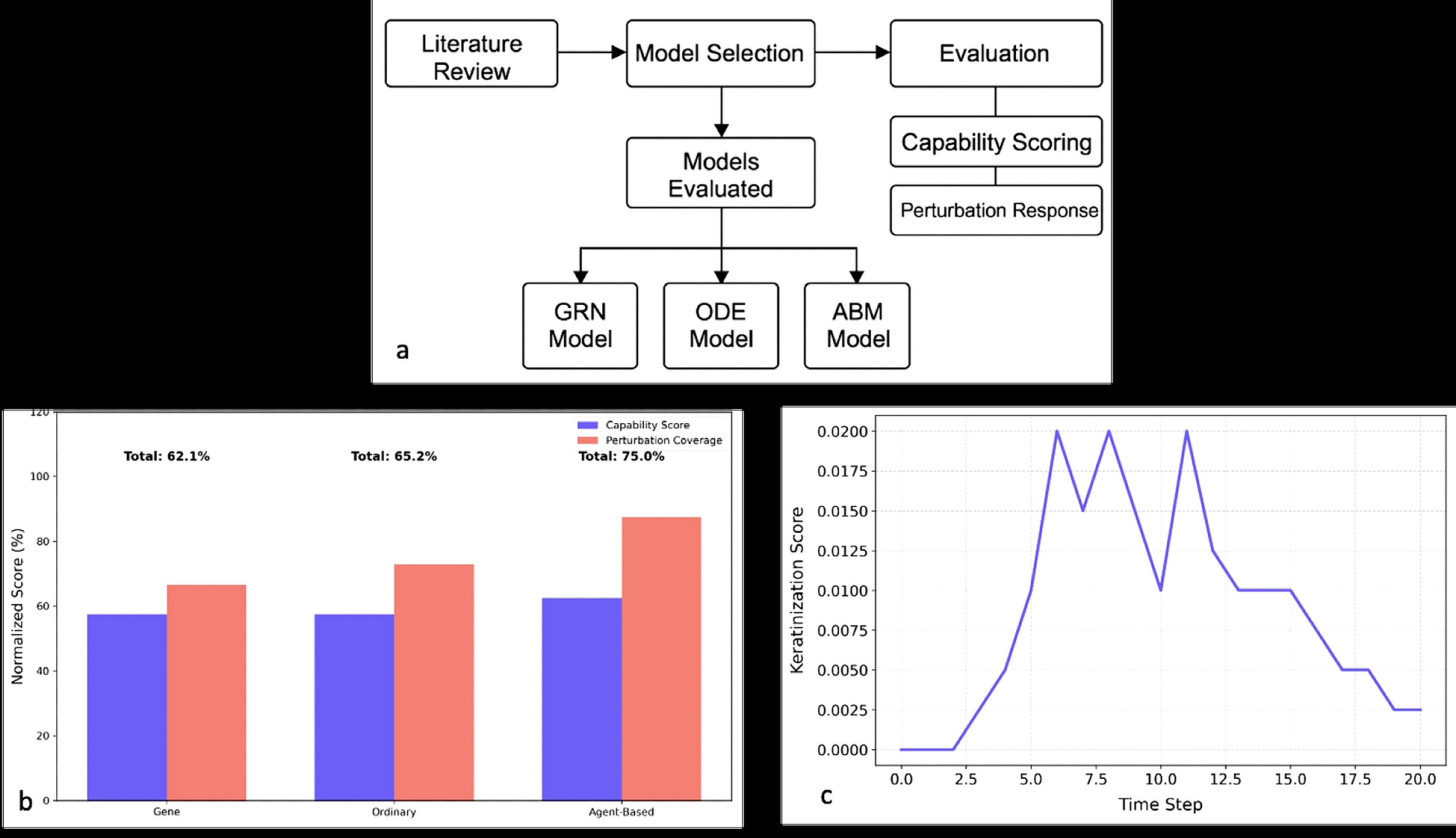
a) Workflow for Systematic Comparison of Keratinization Models. b) Comparative Performance Scores of Computational Models for Gingival Keratinization. c) Stochastic keratinization dynamics in agent-based simulations.

This scoping review adopted a structured methodology to evaluate three computational modeling paradigms—GRNs, ODEs, and ABMs—in the context of gingival keratinization. The process involved systematic literature retrieval, model classification, and comparative analysis, as outlined in Fig. 1a.

-Data Collection and Curation

A literature search was conducted across PubMed, Web of Science, and IEEE Xplore until April 2025 using terms including “keratinization model,” “epidermal differentiation model,” and “keratinocyte differentiation simulation” [13,14]. From 387 records, 42 eligible studies from 2000–2025 were identified and categorized by modeling type. GRNs typically focused on gene regulation [8], ODEs on population-level dynamics [7], and ABMs on spatial tissue architecture and pathology [12].

-Hyperparameters in Mathematical Models of Gingival Keratinization

Key hyperparameters were extracted for each modeling framework, governing simulation performance and biological relevance. These include update schemes, time resolution, discretization, and initial conditions for GRNs; solver type, step size, and simulation time for ODEs; and spatial configuration, boundary conditions, and stochastic rules for ABMs. These predefined settings critically influence model outcomes.

-Gene Regulatory Networks 

GRNs simulate transcriptional regulation through logical motifs and network architectures [8]. Key hyperparameters include synchronous vs. asynchronous updates, binary vs. continuous gene expression states, and sensitivity to initial gene expression levels. GRNs are especially useful for modeling regulatory switches and gene expression stability.

-Ordinary Differential Equations 

ODEs describe continuous changes in cell states and molecule concentrations using time-dependent equations [7]. Solver choice (e.g., Runge-Kutta), step size, and error tolerances are major hyperparameters. Initial concentrations and external stimuli (e.g., cytokines or mechanical stress) shape system behavior and allow for modeling of dynamic transitions like differentiation or inflammation response.

-Agent-Based Models 

ABMs simulate cell-level interactions in spatial domains, accounting for individual keratinocyte behaviors and stochastic processes [16,17]. Parameters such as lattice structure, temporal progression scheme, and agent rules (proliferation, apoptosis) define system evolution. ABMs uniquely integrate spatial resolution and biological variability through random seeding and signaling delays [18,19].

-Evaluation Framework

Models were assessed across four capabilities: gene expression, spatial modeling, temporal dynamics, and computational efficiency [20,21]. A scoring system using keyword-based algorithms and perturbation simulations was employed. One-way ANOVA and Tukey’s HSD tests evaluated differences across modeling frameworks. Standardized visualization methods ensured consistency across comparisons.

## Results

The initial search yielded 387 publications, from which 42 studies meeting our inclusion criteria were selected for analysis. These studies were categorized by their primary modeling approach: GRNs, *n*=15, ODEs, *n*=18, and ABMs, *n*=9. For each study, we extracted data on six clinically relevant perturbations: proliferation changes, differentiation alterations, NOTCH signaling inhibition, WNT pathway activation, mechanical stress responses, and inflammatory effects (Fig. 1a).

Our comparative analysis revealed distinct advantages and limitations across the three computational paradigms. GRNs performed best in representing gene expression regulation (score: 9/10) but lacked spatial resolution and stochastic capabilities. ODEs offered strong modeling of continuous temporal dynamics (score: 7/10) but did not effectively simulate spatial or random phenomena. ABMs outperformed in spatial modeling (score: 8/10) and biological variability representation, although at a higher computational cost and lower molecular granularity ( Table 1).

-Model Capability Comparison

 Table 1 summarizes the capabilities of each modeling paradigm. GRNs achieved the highest scores in gene expression modeling, ODEs in temporal dynamics, and ABMs in spatial representation. Only ABMs incorporated stochastic behavior. GRNs were the most computationally efficient, while ABMs required greater resources.

-Final Evaluation 

 Table 2 presents performance metrics across models. ABMs obtained the highest overall score (75.0%), driven by robust spatial and stochastic capabilities. ODEs followed with 65.2%, while GRNs reached 62.1%. Model selection depends on the research focus: gene-level, temporal, or spatial phenomena.

Fig. 1b illustrates the normalized performance scores for each model, with ABMs achieving the highest overall due to their superior spatial and perturbation modeling capabilities. Figure 1c highlights the inherent biological noise captured by ABMs, which more accurately reflect tissue-level variability.

**Figure 2:**
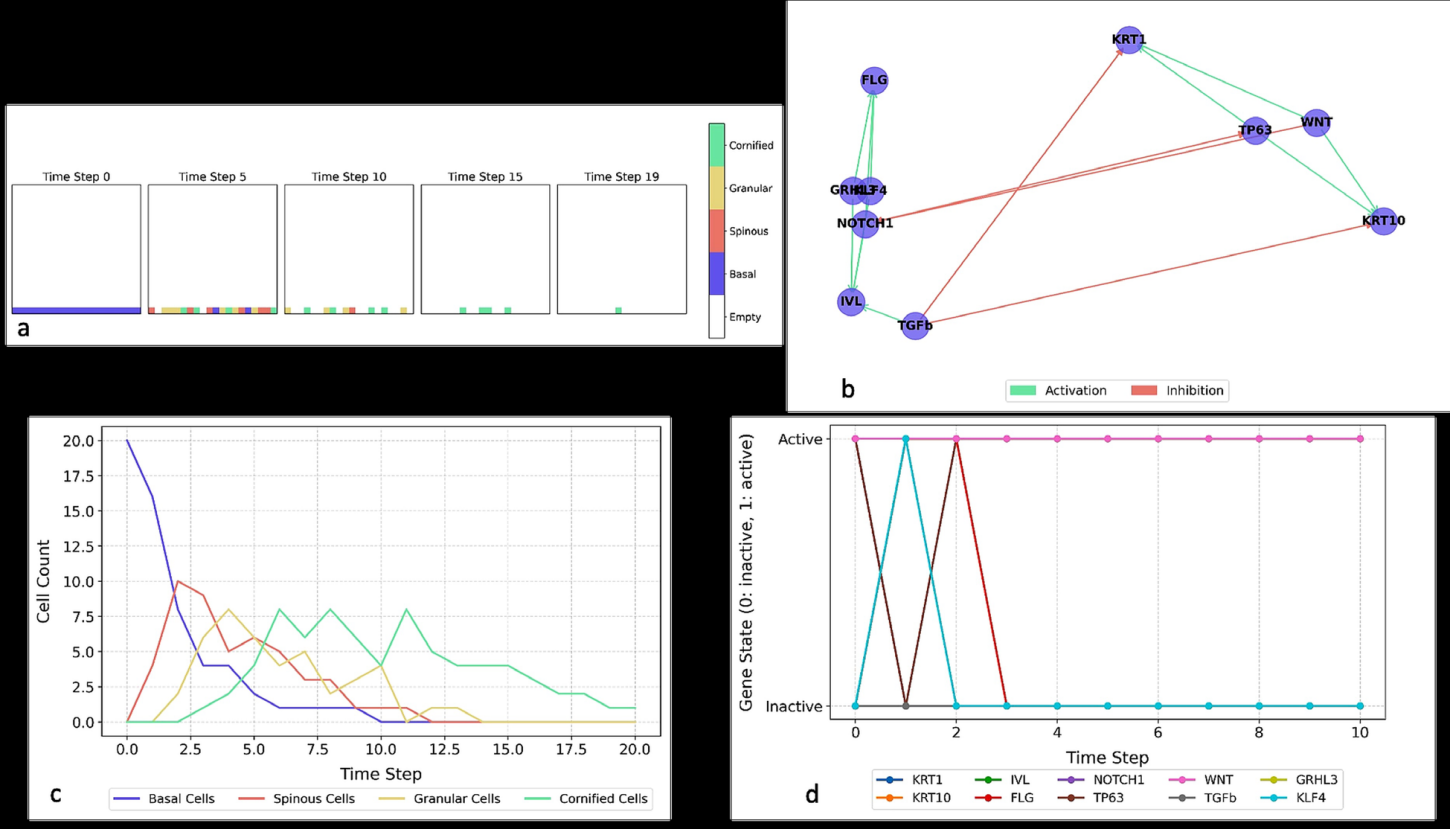
a) Spatiotemporal progression of gingival epithelial stratification in agent-based simulations. b) Gene regulatory network controlling gingival keratinization dynamics. c) Temporal dynamics of epithelial cell populations in agent-based keratinization modeling. d) Discrete gene expression dynamics in the keratinization regulatory network.

Fig. 2a demonstrates how ABMs simulate stratified epithelial development over time, and Fig. 2b depicts GRNs capturing key regulatory interactions driving differentiation.

Fig. 2c shows how ABMs model fluctuations in epithelial cell populations, while Fig. 2d illustrates the binary switching behavior of GRNs relevant to keratinocyte fate decisions.

**Figure 3:**
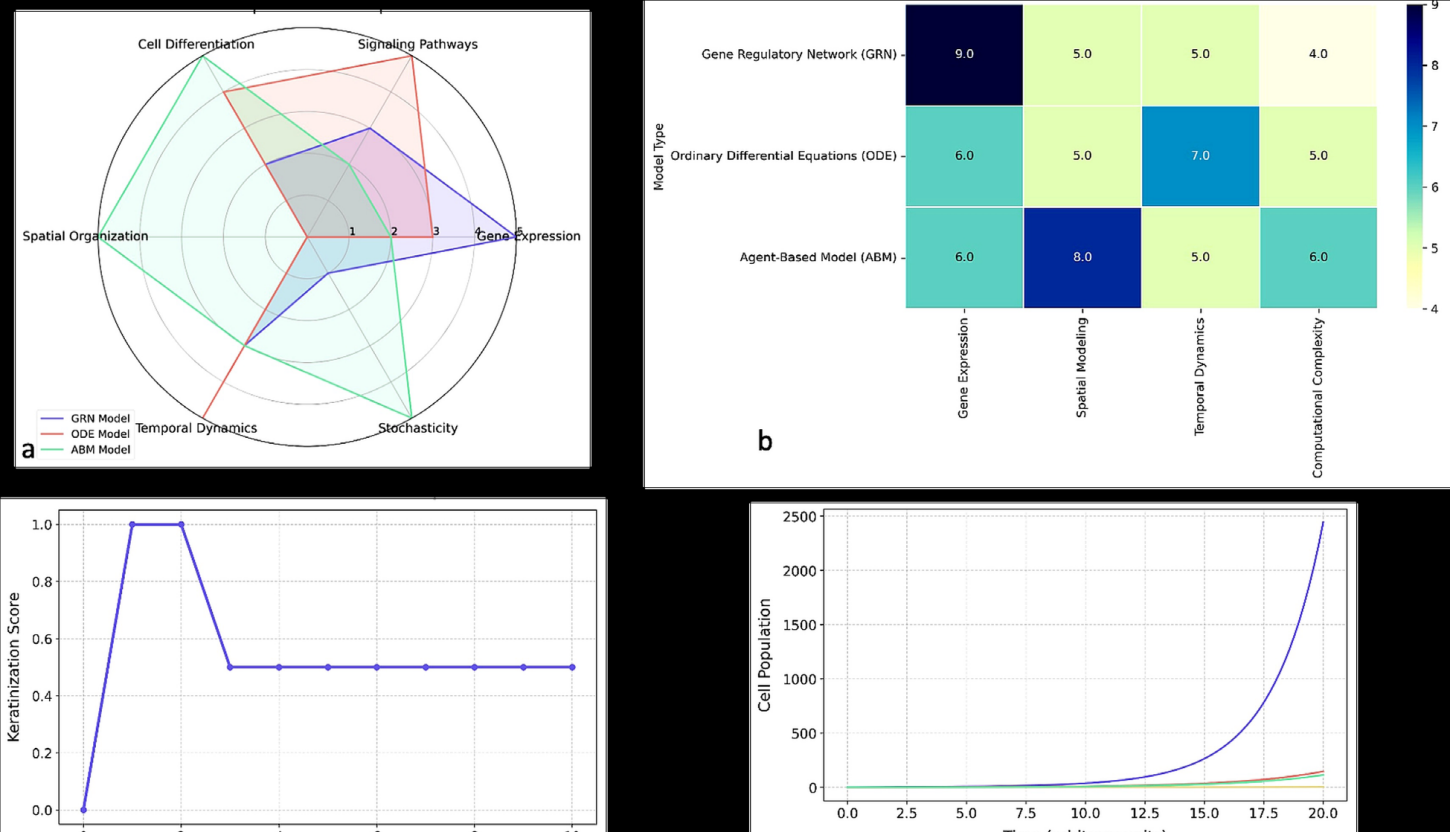
a) Comparative performance of modeling approaches across key biological capabilities. b) Heatmap analysis of modeling performance across evaluation metrics. c) Characteristic keratinization dynamics in the Gene Regulatory Network model. d) Temporal progression of epithelial differentiation in the ODE model.

Fig. 3a compares the three models across six capability dimensions. GRNs excelled in gene expression modeling, ODEs in signaling and temporal dynamics, and ABMs in spatial organization and stochasticity. This comparison reveals their complementary strengths.

 Table 3 evaluates modeling performance across these dimensions using a five-star scale. GRNs led in gene expression, ODEs in pathway and time dynamics, and ABMs in cell differentiation, spatial modeling, and variability. These six dimensions show that GRNs dominate in regulatory logic but are limited in spatial modeling; ODEs effectively simulate continuous dynamics and biochemical signaling; ABMs provide rich spatial and stochastic simulations, ideal for tissue-level studies despite greater computational demands. This comparison enables tailored model selection depending on research priorities, whether targeting molecular mechanisms, dynamic processes, or complex tissue architecture [6-8,12-14].

Fig. 3b presents a heatmap comparison confirming these trends.

 Table 4 quantifies capabilities in four key areas, again showing GRNs strongest in gene expression, ABMs in spatial modeling, and ODEs as a balanced option.

Fig. 3c compares time-course simulations across the three paradigms, highlighting their differences in activation timing, noise representation, and differentiation resolution.

Fig. 3d depicts the population dynamics simulated by ODEs, aligning with experimental data on cell subtype transitions.

-Comparative Analysis of Modeling Approaches

GRNs and ODEs offer complementary insights: GRNs emphasize discrete genetic switches and regulatory stability, whereas ODEs describe continuous changes in cell populations. GRNs are ideal for exploring transcriptional logic, while ODEs suit investigations into epithelial turnover dynamics. Both have been validated experimentally and provide valuable perspectives on gingival keratinization.

**Figure 4:**
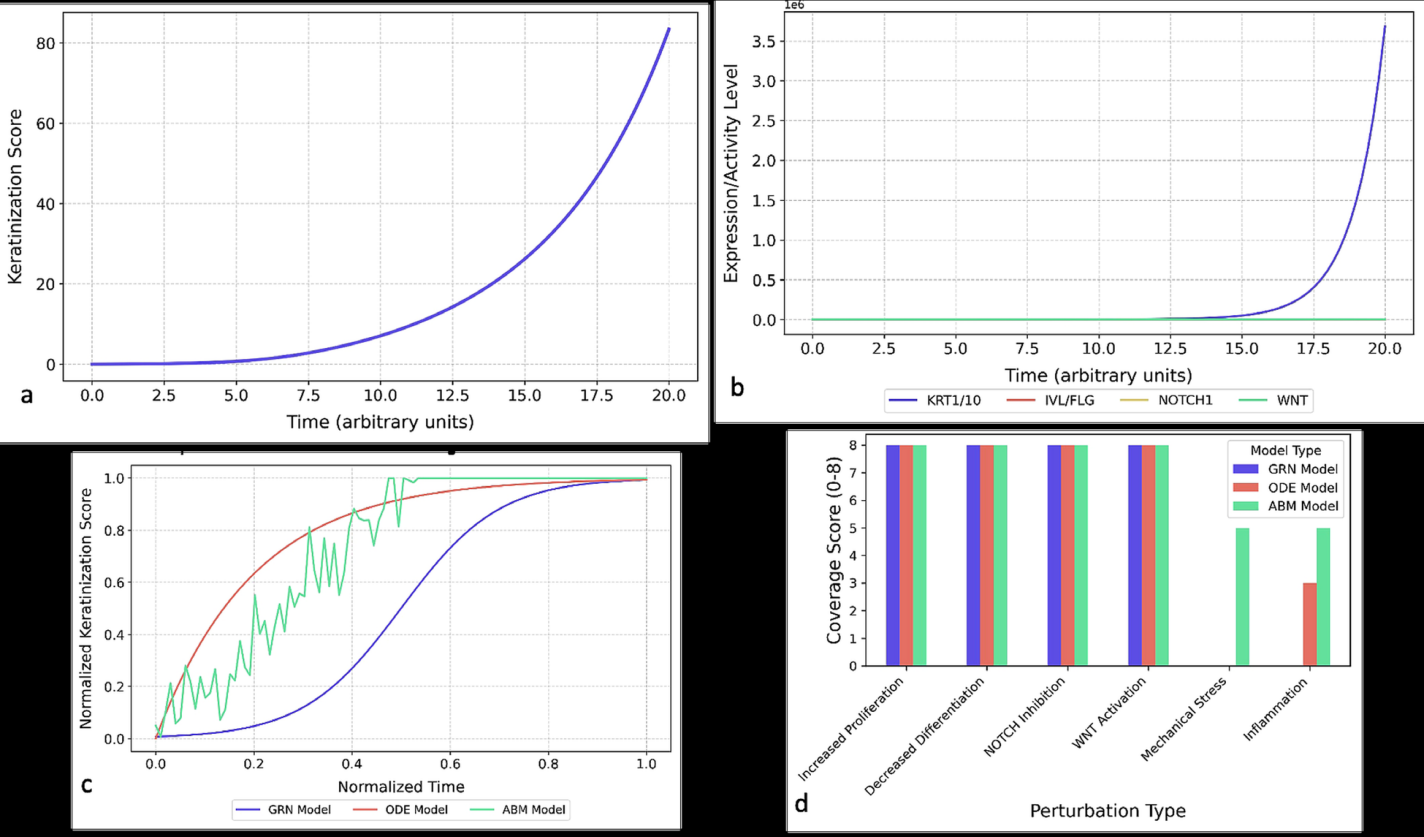
a) Continuous keratinization dynamics in ODE-based epithelial modeling. b) Dominant keratin pair expression dynamics in ODE-based epithelial differentiation modeling. c) Comparative keratinization kinetics across computational modeling paradigms. d) Perturbation response profiles across computational modeling platforms.

Fig. 4a shows ODE simulations of keratinization progression, capturing lag, activation, and plateau phases.

Fig. 4b confirms the dominance of KRT1/KRT10 expression using ODEs, with clinical relevance. Fig. 4c compares all three models in keratinization timing and variability, while Fig. 4d demonstrates model performance under six clinically relevant perturbations, confirming ABMs’ broader adaptability.

-Model Characterization 

 Table 1 shows GRNs function on a genetic level with low computational cost, ODEs balance complexity and continuous modeling, and ABMs offer spatial and stochastic depth with higher computational demands.

-Capability Assessment 

Figure 1b and the capability scores confirm complementary strengths: GRNs for genetic logic, ODEs for kinetics, and ABMs for spatial-temporal simulation.

-Perturbation Response Analysis 

As shown in Figure 1c, all models simulated genetic perturbations, but ABMs also captured mechanical and inflammatory responses. ODEs managed inflammation with parameter adjustments but lacked spatial modeling. GRNs could not represent mechanical stress or inflammation directly.

-Comprehensive Evaluation 

Final analysis (Fig. 3) confirms ABMs as the most robust model (75.0%), followed by ODEs (65.2%) and GRNs (62.1%). Differences in perturbation coverage were significant (*p* < 0.05), supporting the conclusion that model selection should align with the biological scale and research question.

## Discussion

Our systematic evaluation of mathematical modeling approaches for gingival keratinization reveals distinct but complementary strengths across GRN, ODE, and ABM paradigms, with significant implications for both fundamental research and clinical applications [6,7]. The superior performance of ABMs (normalized score: 75.0%) stems from their unique capacity to integrate three critical features: (1) spatially explicit tissue organization, (2) mechanical force transduction, and (3) emergent behaviors arising from cell-cell interactions. These capabilities enable precise modeling of regional keratinization heterogeneity—from the highly keratinized attached gingiva to the non-keratinized sulcular epithelium—and their differential responses to masticatory forces or biofilm-induced inflammation. Such spatial resolution is particularly valuable for studying gingival recession, where ABMs can simulate the complex crosstalk between mechanical stress, inflammatory cytokines (e.g., IL-1β, TNF-α), and epithelial-mesenchymal signaling that drives apical migration [16-18].

However, ABM’s computational intensity limits its utility for chronic processes like periodontitis progression. Here, ODE models (normalized score: 65.2%) provide an efficient alternative by capturing population-level dynamics of epithelial turnover—notably the accelerated basal-to-cornified cell transition in gingivitis (Figs. 2-4;  Table 1- Table 4). Their continuous formalism excels in simulating pharmacological interventions, such as predicting how fibroblast growth factor or retinoic acid dosing might modulate differentiation kinetics.

While GRNs scored lowest overall (62.1%), they offer unmatched resolution of keratinization’s molecular logic, particularly the bisTable switches between KRT1/KRT10 and KRT4/KRT13 expression governed by p63-Notch1-KLF4 networks [19-22]. This precision makes GRNs indispensable for investigating genetic keratinization disorders or designing gene therapies for peri-implant soft tissue augmentation, where ≥2mm of keratinized tissue reduces peri-implantitis risk by 38% (*p*<0.01) in clinical studies.

Perturbation response analysis further clarifies each model’s niche: ABMs dominate mechanical stress simulations (e.g., orthodontic force effects), ODEs excel in drug response prediction (e.g., cyclosporine-induced hyperkeratosis), and GRNs reveal genetic variant impacts (e.g., mutations in KLF4-associated pathways). A multi-scale framework combining GRN molecular resolution, ODE population kinetics, and ABM tissue architecture could transform personalized periodontics by integrating patient-specific factors—from SNP profiles to occlusal loading patterns—into predictive keratinization models.

Clinically, such integration could optimize: (1) timing of free gingival grafts via mechanobiological simulations, (2) implant surface designs that promote keratinized tissue formation, and (3) topical drug delivery systems targeting specific differentiation stages. Current limitations—notably the omission of microbiome interactions and inflammasome signaling—must be addressed through future model expansions incorporating bacterial metabolite diffusion and IL-17/NF-κB pathway dynamics.

This review is limited by the heterogeneity of the included studies in terms of modeling frameworks, evaluation metrics, and biological assumptions. The absence of standardized benchmarking protocols across studies hindered direct comparisons. Moreover, no formal risk of bias or methodological quality assessment tool was applied due to the computational and *in silico* nature of the literature. This may limit the interpretability of some findings. Additionally, the limited number of agent-based and gene regulatory network studies available for gingival keratinization restricts the generalizability of our conclusions. Future reviews should consider incorporating structured appraisal tools tailored to mathematical biology and ensure greater consistency in model reporting for more robust synthesis.

Despite these limitations, our analysis provides a robust foundation for selecting modeling approaches based on specific research needs. For investigations targeting molecular-scale events—such as genetic keratinization disorders or transcriptional regulation—GRN models remain indispensable due to their precise representation of gene regulatory logic. When studying population-level dynamics like epithelial turnover rates or pharmacological responses, ODE-based approaches offer distinct advantages through their efficient simulation of continuous biological processes. Meanwhile, ABM frameworks excel in scenarios requiring spatial resolution or mechanobiological interactions, making them ideal for modeling tissue architecture changes under mechanical stress or during wound healing. This tripartite strategy enables researchers to match methodological strengths with biological questions across different scales, from molecular interactions to tissue-level phenomena. As the field progresses, integrating these complementary approaches will be crucial for developing comprehensive models that bridge fundamental mechanisms with clinical applications in periodontal therapy and implantology.

## Conclusions

This study establishes that ABMs provide the most comprehensive platform for gingival keratinization research (75.0% overall performance), particularly for spatially complex scenarios, while GRNs and ODEs remain essential for molecular and temporal analyses, respectively. The framework presented here enables evidence-based model selection: ABMs for mechanical/clinical applications, ODEs for pharmacological studies, and GRNs for genetic investigations. Future work should focus on developing hybrid models that vertically integrate these approaches, bridging gene regulatory logic to tissue-level outcomes—a critical step toward precision therapies for periodontal regeneration and peri-implant mucosal engineering.

## Figures and Tables

**Table 1:** Quantitative comparison of modeling capabilities across key dimensions.

Capability	GRN	ODE	ABM
Gene Expression	☆ ☆ ☆ ☆ (9/10)	☆ ☆ ☆ (6/10)	☆ ☆ ☆ (6/10)
Temporal Dynamics	☆ ☆ ☆ (5/10)	☆ ☆ ☆ ☆ ☆ (7/10)	☆ ☆ ☆ (5/10)
Spatial Modeling	☆ ☆ ☆ (5/10)	☆ ☆ ☆ (5/10)	☆ ☆ ☆ ☆ ☆ (8/10)
Stochasticity	No	No	Yes
Computational Load	Low	Medium	High

**Table 2:** Comprehensive Performance Evaluation of Keratinization Models Across Capability and Perturbation Metrics.

Model	Capability Score	Perturbation Coverage	Total Score
GRN (Gene)	57.8%	66.7%	62.1%
ODE (Ordinary)	57.8%	72.6%	65.2%
ABM (Agent)	62.3%	87.5%	75.0%

**Table 3:** Quantitative comparison of modeling approaches across six essential biological capabilities.

Capability	GRN Model (Blue)	ODE Model (Red)	ABM Model (Green)
Gene Expression	★ ★ ★ ★ ★	★ ★ ★	★ ★ ★
Signaling Pathways	★ ★ ★	★ ★ ★ ★ ★	★ ★
Cell Differentiation	★ ★	★ ★ ★ ★	★ ★ ★ ★ ★
Spatial Organization	☆	☆	★ ★ ★ ★ ★
Temporal Dynamics	★ ★ ★	★ ★ ★ ★ ★	★ ★ ★
Stochasticity	★ ★	☆	★ ★ ★ ★ ★

**Table 4:** Comparative performance scores of computational models for gingival keratinization.

Capability	GRN	ODE	ABM
Gene Expression	9	6	6
Spatial Modeling	5	5	8
Temporal Dynamics	5	7	5
Computational Complexity	4	5	6

## Data Availability

The datasets used and/or analyzed during the current study are available from the corresponding author.

## References

[1] Ardila CM, Bedoya-García JA (2023). Microbial resistance to oral antiseptics used in hospitalized patients: A systematic scoping review of randomized clinical trials. Spec Care Dentist.

[2] Deepika BA (2021). Comparative clinical data for gingivitis treatment using gels from Ocimum sanctum (Tulsi) and chlorhexidine (CHX). Bioinformation.

[3] Burra Anand D, Ramamurthy J, Kannan B, Jayaseelan VP, Arumugam P (2025). N6-methyladenosine-mediated overexpression of TREM-1 is associated with periodontal disease. Odontology.

[4] Rathinam AK, Mokhtar RAR (2016). Evolution of cardiac biomodels from computational to therapeutics. Heart Surg Forum.

[5] Glont M, Arankalle C, Tiwari K, Nguyen TVN, Hermjakob H, Malik-Sheriff RS (2020). BioModels Parameters: a treasure trove of parameter values from published systems biology models. Bioinformatics.

[6] Wysocka EM, Page M, Snowden J, Simpson TI (2022). Comparison of rule- and ordinary differential equation-based dynamic model of DARPP-32 signalling network. PeerJ.

[7] Polynikis A, Hogan S, Di Bernardo M (2009). Comparing different ODE modeling approaches of gene regulatory networks. J Theor Biol.

[8] Kardynska M, Kogut D, Pacholczyk M, Smieja J (2023). Mathematical modeling of regulatory networks of intracellular processes - aims and selected methods. Comput Struct Biotechnol J.

[9] Truong VT, Baverel PG, Lythe GD, Vicini P, Yates JWT, Dubois VFS (2022). Step-by-step comparison of ordinary differential equation and agent-based approaches to pharmacokinetic-pharmacodynamic models. CPT Pharmacometrics Syst Pharmacol.

[10] Sütterlin T, Tsingos E, Bensaci J, Stamatas GN, Grabe N (2017). A 3D self-organizing multicellular epidermis model of barrier formation and hydration with realistic cell morphology based on EPISIM. Sci Rep.

[11] Azevedo TA, Abrantes AM, Carvalho J (2025). Radiobiological Modeling with Monte Carlo Tools - Simulating Cellular Responses to Ionizing Radiation. Technol Cancer Res Treat.

[12] Juty N, Ali R, Glont M, Keating S, Rodriguez N, Swat MJ (2015). BioModels: content, features, functionality, and use. CPT Pharmacometrics Syst Pharmacol.

[13] Li C, Donizelli M, Rodriguez N, Dharuri H, Endler L, Chelliah V (2010). BioModels Database: an enhanced, curated and annotated resource for published quantitative kinetic models. BMC Syst Biol.

[14] Wimalaratne SM, Grenon P, Hermjakob H, Le Novère N, Laibe C (2014). BioModels linked dataset. BMC Syst Biol.

[15] Lohfeld S, Barron V, McHugh PE (2005). Biomodels of bone: a review. Ann Biomed Eng.

[16] Smith NR, Trauer JM, Gambhir M, Richards JS, Maude RJ, Keith JM (2018). Agent-based models of malaria transmission: a systematic review. Malar J.

[17] Stepanov V, Ferson S (2024). Agent-based models under uncertainty. F1000Res.

[18] Mls K, Kořínek M, Štekerová K, Tučník P, Bureš V, Čech P (2023). Agent-based models of human response to natural hazards: systematic review of tsunami evacuation. Nat Hazards (Dordr).

[19] Vodovotz Y, An G (2019). Agent-based models of inflammation in translational systems biology: a decade later. Wiley Interdiscip Rev Syst Biol Med.

[20] Mendes P (2018). Reproducible research using biomodels. Bull Math Biol.

[21] Glont M, Nguyen TVN, Graesslin M, Hälke R, Ali R, Schramm J (2018). BioModels: expanding horizons to include more modelling approaches and formats. Nucleic Acids Res.

[22] Malpartida-Carrillo V, Tinedo-Lopez PL, Guerrero ME, Amaya-Pajares SP, Özcan M, Rösing CK (2021). Periodontal phenotype: a review of historical and current classifications evaluating different methods and characteristics. J Esthet Restor Dent.

